# Neural correlates of shared sensory symptoms in autism and attention-deficit/hyperactivity disorder

**DOI:** 10.1093/braincomms/fcaa186

**Published:** 2020-11-02

**Authors:** Takashi Itahashi, Junya Fujino, Taku Sato, Haruhisa Ohta, Motoaki Nakamura, Nobumasa Kato, Ryu-Ichiro Hashimoto, Adriana Di Martino, Yuta Y Aoki

**Affiliations:** 1 Medical Institute of Developmental Disabilities Research, Showa University, Tokyo 157-8577, Japan; 2 Department of Language Sciences, Graduate School of Humanities, Tokyo Metropolitan University, Tokyo, Japan; 3 Autism Center, Dr John and Consuela Phelan Scholar, Child Mind Institute, New York, NY, USA

**Keywords:** ADHD, ASD, functional connectivity, research domain criteria, sensory symptoms

## Abstract

Symptoms of autism spectrum disorder and attention-deficit/hyperactivity disorder often co-occur. Among these, sensory impairment, which is a core diagnostic feature of autism spectrum disorder, is often observed in children with attention-deficit/hyperactivity disorder. However, the underlying mechanisms of symptoms that are shared across disorders remain unknown. To examine the neural correlates of sensory symptoms that are associated with autism spectrum disorder and attention-deficit/hyperactivity disorder, we analysed resting-state functional MRI data obtained from 113 people with either autism spectrum disorder or attention-deficit/hyperactivity disorder (*n *=* *78 autism spectrum disorder, mean age = 29.5; *n *=* *35 attention-deficit/hyperactivity disorder, mean age = 31.2) and 96 neurotypical controls (mean age = 30.6, range: 20–55 years) using a cross-sectional study design. First, we used a multi-dimensional approach to examine intrinsic brain functional connectivity related to sensory symptoms in four domains (i.e. low registration, sensation seeking, sensory sensitivity and sensation avoidance), after controlling for age, handedness and head motion. Then, we used a partial least squares correlation to examine the link between sensory symptoms related to intrinsic brain functional connectivity and neurodevelopmental symptoms measured using the Autism Spectrum Quotient and Conners’ Adult Attention-Deficit/Hyperactivity Disorder Rating Scale, regardless of diagnosis. To test whether observed associations were specific to sensory symptoms related to intrinsic brain functional connectivity, we conducted a control analysis using a bootstrap framework. The results indicated that transdiagnostic yet distinct intrinsic brain functional connectivity neural bases varied according to the domain of the examined sensory symptom. Partial least squares correlation analysis revealed two latent components (latent component 1: *q *<* *0.001 and latent component 2: *q *<* *0.001). For latent component 1, a set of intrinsic brain functional connectivity was predominantly associated with neurodevelopmental symptom-related composite score (*r *=* *0.64, *P *<* *0.001), which was significantly correlated with Conners’ Adult Attention-Deficit/Hyperactivity Disorder Rating Scale total *T* scores (*r *=* *−0.99, *q *<* *0.001). For latent component 2, another set of intrinsic brain functional connectivity was positively associated with neurodevelopmental symptom-related composite score (*r *=* *0.58, *P *<* *0.001), which was eventually positively associated with Autism Spectrum Quotient total scores (*r *=* *0.92, *q *<* *0.001). The bootstrap analysis showed that the relationship between intrinsic brain functional connectivity and neurodevelopmental symptoms was relative to sensory symptom-related intrinsic brain functional connectivity (latent component 1: *P *=* *0.003 and latent component 2: *P *<* *0.001). The current results suggest that sensory symptoms in individuals with autism spectrum disorder and those with attention-deficit/hyperactivity disorder have shared neural correlates. The neural correlates of the sensory symptoms were associated with the severity of both autism spectrum disorder and attention-deficit/hyperactivity disorder symptoms, regardless of diagnosis.

## Graphical Abstract

**Graphical Abstract:**
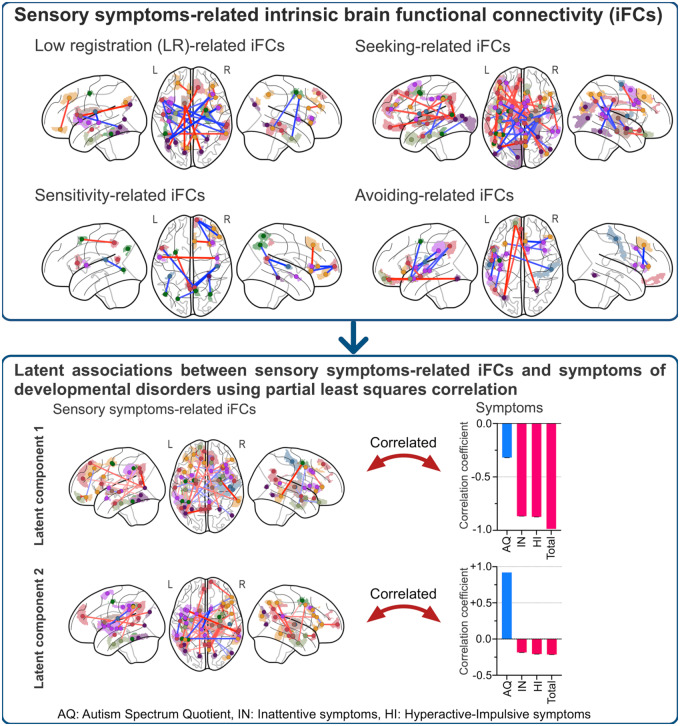

## Introduction

Autism spectrum disorder (ASD) is characterized by social communication impairment (SCI) along with restricted interests and repetitive behaviours, while attention-deficit/hyperactivity disorder (ADHD) is classified according to age-inappropriate inattention, hyperactivity and impulsivity (American Psychiatric Association, 2013). Symptoms of ASD and ADHD often co-occur (Grzadzinski *et al.*, 2011), including sensory symptoms (Ghanizadeh, 2011; Bijlenga *et al.*, 2017; Little *et al.*, 2018). These generally involve hyper- or hypo-reactivity to sensory inputs, which is one of the four restricted interests and repetitive behaviours diagnostic features of ASD as defined by the Diagnostic and Statistical Manual of Mental Disorders fifth edition (DSM-5). This domain was not included in the DSM-IV diagnostic criteria (American Psychiatric Association, 2000). Sensory symptoms in individuals with ASD and ADHD have increasingly been a research focus because of their high prevalence (Crane *et al.*, 2009), as well as their potential link with other cognitive domains [reviewed by Baranek *et al.* (2018) and Thye *et al.* (2018)]. Theoretically, hyper-reactivity to sensory input can make it more difficult to focus on social stimuli, while hypo-reactivity can decrease learning opportunities. Thus, both hyper- and hypo-reactivity may be related to SCI and inattention (Baranek *et al.*, 2018; Thye *et al.*, 2018). Indeed, prior studies have demonstrated that sensory symptoms are associated with language skills and adaptive behaviour in individuals with ASD (Lane *et al.*, 2010; Watson *et al.*, 2011).

Atypical intrinsic brain functional connectivity (iFC) is a well-known element of ASD pathophysiology (Shen *et al.*, 2016). Supporting the theory that sensory symptoms contribute to symptoms in other domains, resting-state functional MRI studies have demonstrated the importance of the sensory cortex in ASD symptoms. For example, in individuals with ASD, iFC between the primary sensory cortex and other brain regions was found to be atypical (Oldehinkel *et al.*, 2019) and was correlated with ASD symptoms (Cerliani *et al.*, 2015). Further, a novel approach showed that stepwise functional connectivity between the primary sensory cortex and transmodal association cortices was associated with SCI severity (Hong *et al.*, 2019; Martinez *et al.*, 2020). However, the neural mechanisms of the hierarchical relationships between sensory and SCI symptoms remain unknown.

Like ASD, ADHD is characterized by atypical iFCs [reviewed by Castellanos and Aoki (2016)]. While categorical comparisons of iFCs between individuals with ASD and ADHD have yielded both shared and distinct patterns (Ray *et al.*, 2014; Dajani *et al.*, 2019), studies that accounted for comorbidity and used a multi-dimensional approach have provided a more complete picture. Specifically, atypical iFC patterns in individuals with ADHD are similar to those in people with ASD with comorbid ADHD symptoms. This suggests that the effects of ADHD symptoms on iFC are, in part, comparable in individuals with ASD and those with ADHD (Di Martino *et al.*, 2013). Diffusion tensor imaging has revealed similarities between ASD and ADHD (Ameis *et al.*, 2016), as well as a continuous distribution of structural connectivity-ASD symptom relationships across individuals with ASD and those with ADHD (Aoki *et al.*, 2017). The findings of these functional and structural connectivity studies support the notion that brain–behaviour relationships transcend diagnostic boundaries across these two disorders. However, no studies have dissected the symptom domains of either ASD or ADHD, and so the specific role of sensory impairment across diagnoses remains unknown.

In theory, sensory symptoms may lead to both SCI and inattention symptoms (Baranek *et al.*, 2018; Thye *et al.*, 2018). Indeed, atypical function of sensory processing pathways is associated with the pathophysiology of SCI symptoms in people with ASD (Cerliani *et al.*, 2015; Hong *et al.*, 2019; Oldehinkel *et al.*, 2019). Both SCI and sensory symptoms are also observed in individuals with ADHD (Ghanizadeh, 2011; Grzadzinski *et al.*, 2011; Bijlenga *et al.*, 2017; Little *et al.*, 2018). Indeed, the neural correlates for ASD symptoms may transcend the diagnostic boundary between ASD and ADHD (Di Martino *et al.*, 2013; Aoki *et al.*, 2017). Thus, it may be helpful to investigate the neural mechanisms underlying the hierarchical relationships between sensory and SCI symptoms, regardless of diagnosis.

The aim of the current study can be interpreted in the context of the research domain criteria (Insel *et al.*, 2010). Prior biological studies with case–control designs have produced inconsistent and non-disorder-specific findings. Thus, investigating hierarchical relationships between symptoms along with their neural bases, regardless of diagnosis, may represent a way to focus on biological pathophysiology as opposed to diagnosis. In the current study, we hypothesized that sensory symptoms would be associated with iFC that is correlated with symptoms of ASD, especially those in the SCI domain, regardless of diagnosis. To test this hypothesis, we first identified the iFC that was transdiagnostically associated with sensory symptoms. Then, we examined whether sensory symptom-related iFC was associated with Autism Spectrum Quotient (AQ) and/or Conners’ Adult ADHD Rating Scale (CAARS) scores. Finally, we tested whether the relationships between iFC and neurodevelopmental symptoms (i.e. AQ and/or CAARS scores) were specific to sensory symptom-related iFCs. The analyses were conducted across a group of participants with ASD, ADHD and neurotypical controls (NTCs).

## Materials and methods

### Participants

In this cross-sectional study, we obtained MRI data from 266 adults (20–55 years old) who either had ASD, ADHD or were NTCs. All individuals agreed to undergo MRI scanning and were recruited at Showa University Karasuyama Hospital between September 2014 and March 2019. The dataset passed a visual inspection for artefacts and macro-brain anomalies. Fifteen participants were excluded because of excessive motion (five ASD, two ADHD and eight NTC; see below). Originally, there was a male predominance in the sample. To increase the biological homogeneity of the sample, we excluded female participants (42 women). As a result, we analysed MRI data from 78 men with primary ASD, 35 men with primary ADHD and 96 male NTCs (Supplementary Fig. 1). A clinical team assessed developmental history, present illness, life history and family history, and then made clinical diagnoses based on the DSM-IV-TR. The first or second edition of the Autism Diagnostic Observation Schedule (ADOS) was used depending on whether the participant had enrolled before or after publication of the second edition (Gotham *et al.*, 2007, 2009). A reliable trained psychologist administered Module 4 of the ADOS to 57 participants with ASD and 10 participants with ADHD. Thirty-three individuals whose primary diagnosis was ASD were taking medication (19 were taking antidepressants, 18 were taking benzodiazepine and two were taking psychostimulants), and 24 individuals with ADHD were taking psychoactive medication (22 were taking psychostimulants, four were taking antidepressants and two were taking benzodiazepine). The NTCs were confirmed to have no psychiatric conditions according to the Japanese version of the Mini-International Neuropsychiatric Interview (Sheehan *et al.*, 1998). The intelligence quotient scores of participants with ASD and those with ADHD were estimated using the Wechsler Adult Intelligence Scale-Third Edition or the WAIS-Revised, while those for the NTCs were estimated using a Japanese version of the National Adult Reading Test (Matsuoka *et al.*, 2006). The AQ has five subscales: social skills, communication skills, imagination, attention to detail and attention switching/tolerance of change (Baron-Cohen *et al.*, 2001). Some of the subscales, such as attention to detail and attention switching/tolerance of change, reflect symptoms in the DSM-5. However, in the current participant group, total AQ scores were well correlated with the SCI subdomains (social skills + communication skills) [*r *=* *0.94, 95% confidence interval (CI) = [0.93, 0.96], *P *<* *0.001]. Thus, we utilized the total AQ score as a reflection of SCI severity. ADHD symptoms were evaluated using scores from the CAARS (Conners *et al.*, 1999). All participants completed the Adolescent/Adult Sensory Profile (AASP), which assesses sensory impairments in four domains: low registration, sensation seeking, sensory sensitivity and sensation avoiding (Brown and Dunn, 2002). The four domains of the AASP differ in terms of the ways in which neurological thresholds (i.e. high or low) interact with behavioural responses (i.e. passive or active). Low registration refers to high neurological thresholds and passive behavioural responses, while sensation seeking refers to high neurological thresholds and active behavioural responses. Both sensory sensitivity and sensation avoiding refer to low neurological thresholds while the former and latter denote passive and active behavioural responses, respectively. Here, a person with ‘high neurological thresholds’ would take longer to perceive stimuli or be more likely to miss stimuli compared with a person with low neurological thresholds.

### MRI acquisition

All MRI data were acquired using a 3.0-T MRI scanner (MAGNETON Verio, Siemens Medical Systems, Erlangen, Germany) with a 12-channel head coil. Functional images were acquired using an echo planar imaging sequence (repetition time: 2500 ms, echo time: 30 ms, flip angle: 80°, field of view: 212 mm, matrix size: 64 × 64, slice thickness: 3.2 mm with a 0.8-mm gap, 40 axial slices) at rest for 10 min 10 s (244 volumes). During the resting-state scans, participants were asked to fixate their eyes on a cross-hair displayed at the centre of the screen, not to think about specific things, and to stay awake. To correct for susceptibility-induced distortions of the functional image, gradient echo field mapping images were acquired immediately after the resting-state scans (repetition time: 488 ms, short echo time: 4.92 ms, long echo time: 7.38 ms, flip angle: 60°, field of view: 212 mm, matrix size: 64 × 64, slice thickness: 3.2 mm with a 0.8-mm gap, 40 axial slices). For normalization purposes, T_1_-weighted images were acquired with an MPRAGE sequence (repetition time: 2.3 s, echo time: 2.98 ms, flip angle: 9°, field of view: 256 mm, matrix size: 256 × 256, slice thickness: 1 mm, 240 sagittal slices, voxel size: 1 × 1 × 1 mm).

### Resting-state functional MRI preprocessing and iFC estimation

The resting-state functional MRI data were then preprocessed using FMRIPREP version 1.1.8 (Esteban *et al.*, 2019). Briefly, the first four volumes were removed to allow for T1 equilibration. Preprocessing steps included head motion estimation, slice timing correction, co-registration of echo planar image data to the corresponding T_1_-weighted anatomical image, distortion correction and normalization to standard Montreal Neurological Institute space. Data with a translation (*x*, *y*, *z*) of 3 mm or larger and/or rotation (pitch, yaw and roll) parameters of three degrees or larger were discarded. For more details regarding the preprocessing pipeline, see http://fmriprep.readthedocs.io/en/latest/workflows.html. We analysed the preprocessed data using the Human Connectome Project style surface-based methods with the ciftify toolbox, version 2.1.1 (Dickie *et al.*, 2018). This toolbox allowed us to analyse our non-Human Connectome Project style data using a Human Connectome Project-like surface-based pipeline.

After converting the preprocessed volumetric data to surface-based data, we performed a nuisance regression in a vertex-wise manner. Nuisance signals consisted of linear detrending, six head motion parameters and the average signals from subject-specific white matter, cerebrospinal fluid and the whole brain, with their derivatives. Band-pass filtering (0.008 and 0.1 Hz) was applied to the residuals. Glasser’s 360 surface-based cortical atlas (Glasser *et al.*, 2016) with 16 subcortical parcels was used to identify regions of interest. The subcortical parcels were defined using FreeSurfer and stored in the CIFTI format (Glasser *et al.*, 2013). Frame-wise displacement (FD) was computed to quantify spurious changes caused by head motion. Then, motion-contaminated frames with FD > 0.5 mm were removed. Participants for whom the median FD or the ratio of volumes excluded by scrubbing were 3 standard deviations above the group mean were also excluded. After following these steps, no significant between-group differences were observed in either the median FD (*P *=* *0.95) or the ratio of retained volumes (*P *=* *0.1). Pearson correlations were calculated among all possible pairs of regions of interest, yielding a 376 × 376 iFC matrix for each subject, and Fisher’s *r*-to-*z* transform was applied to iFCs. To facilitate the interpretation of our findings, we identified the anatomical labels of each region of interest and the names of the resting-state networks using automated anatomical labelling and the methods of a previous study (Yeo *et al.*, 2011) in which the spatial overlap was calculated between each region of interest and automated anatomical labelling or network mask.

### Statistical analysis

#### Overview of the analytical procedures used in this study

**Figure 1:**
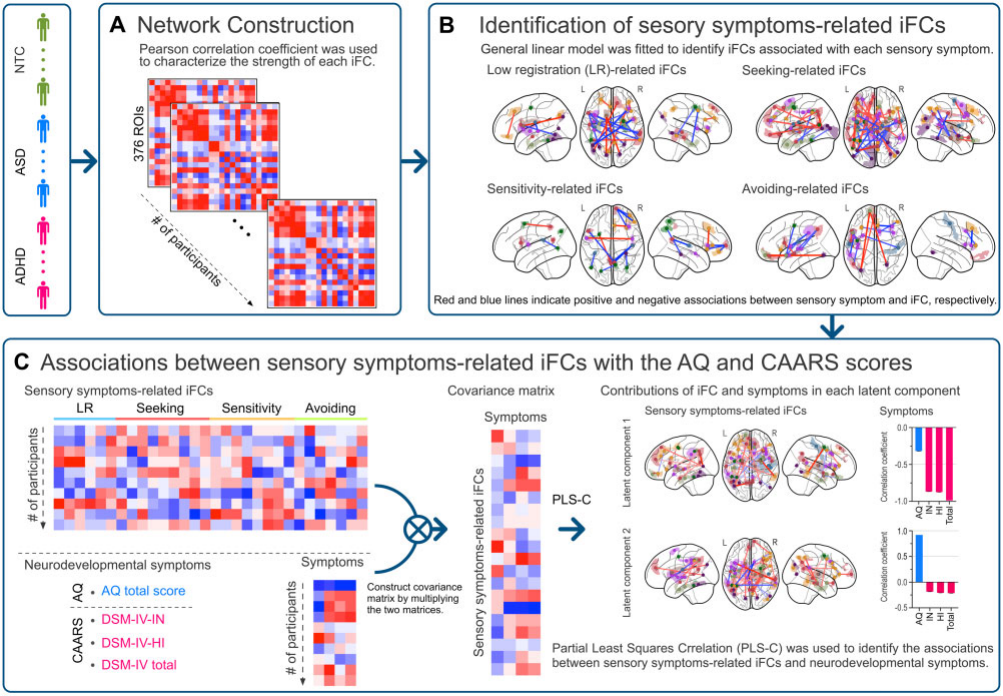
**Overview of study procedure.** In this study, we first constructed an intrinsic brain functional connectivity (iFC) matrix for each participant using Glasser’s cortical atlas (A). Second, a multiple linear regression model was fitted to each matrix to identify iFC associated with four sensory symptoms (i.e. low registration, sensory seeking, sensory sensitivity and sensory avoiding) (B). Then, we applied a multivariate data-driven method called partial least squares correlation (PLS-C) to a set of sensory symptom-related iFC and neurodevelopmental symptoms [i.e. the Autism Spectrum Quotient (AQ) total score and scores on Conners’ Adult ADHD Rating Scale (CAARS)], to investigate the associations between them according to latent components (C).

An overview of the analytical procedures is shown in Fig. 1. First, we constructed an iFC matrix from the resting-state functional MRI data for each participant using Glasser’s cortical atlas. Second, we identified multiple sets of iFCs, each associated with a different aspect of sensory symptoms (i.e. low registration, sensation seeking, sensory sensitivity and sensation avoiding). Third, we applied a data-driven method called partial least squares correlation (PLS-C) to identify the latent components (LCs) that maximally covaried between a set of sensory symptom-related iFCs and the neurodevelopmental symptoms measured by the AQ and CAARS. Finally, we examined the association between iFCs and the neurodevelopmental symptoms specific to the sensory-related iFCs.

#### Identification of iFC associated with the sensory symptoms

We fitted a general linear model as follows:

$$y_{ij}=\beta_{0}+\sum_{l=1}^{4}\beta_{l}x_{il}+\sum_{m=1}^{3}\beta_{m}x_{mi}+\varepsilon_{ij},$$

where *y_ij_* and *ε_ij_* represent the *j*th iFC and residual of the *i*th subject. The first and second terms in the equation correspond to the four sensory symptom severity scores (i.e. low registration, sensation seeking, sensory sensitivity and sensation avoiding) and to the nuisance covariates (i.e. age, handedness and median FD), respectively. This model allowed us to examine the effects of each of the four sensory symptoms, while regressing out the other variables. Permutation tests were performed to calculate *P*-values from null distributions. The number of iterations was set to 5000 in this study. The statistical threshold was set to *P *<* *0.05 after false discovery rate correction, implemented in the Network-Based Statistics toolbox.

#### Sensory symptom-related iFC associations between AQ and CAARS

To investigate the ways in which sensory symptom-related iFC was associated with AQ and CAARS scores across all individuals for whom scores were available, we used PLS-C to investigate statistical associations between two sets of observed variables. Specifically, we identified LCs in one variable set (i.e. sensory symptom-related iFCs) that maximally covaried with LCs in another set (i.e. AQ and CAARS). Since this method linearly projects two sets of observed variables into a common latent space, it yields sensory symptom-related iFC composite scores and neurodevelopmental symptom-related composite scores (i.e. AQ and CAARS scores) for each LC. Sensory symptom-related iFC and neurodevelopmental symptom data are stored in matrices ***X*** and ***Y***, respectively. The number of rows corresponds to the number of participants while the number of columns corresponds to the number of features (i.e. sensory-related iFC or neurodevelopmental symptoms). The effects of sensory symptoms and other confounding factors (i.e. age, handedness and median FD) were regressed out from both matrices, and residualized matrices were *Z*-scored. Then, we computed the covariance matrix ***R*** as follows:

$$R=X^{T}\cdot Y.$$

We used singular value decomposition to decompose the covariance matrix, ***R***, into three matrices:

$$R=U\cdot S\cdot V^{T}.$$

The left singular and right singular vectors are called neurodevelopmental symptom-related and sensory symptom-related iFC *saliences*, respectively, while ***S*** is a diagonal matrix in which diagonal elements are singular values. Next, we calculated sensory symptom-related iFC and neurodevelopmental symptom-related composite scores, ***L_X_*** and ***L_Y_***, by projecting ***X*** and ***Y*** onto their respective saliences:

$$L_{X}=X\cdot U\;\mathrm{and}\;L_{Y}=Y\cdot V.$$

These composite scores reflect sensory symptom-related iFC and the neurodevelopmental symptom-related contribution to each LC, for each individual.

The number of statistically significant LCs was determined by a permutation test. Briefly, we first randomly reordered the rows (i.e. the order of subjects) of ***X*** such that the original association between ***X*** and ***Y*** was destroyed. Then, PLS-C was applied to the permuted dataset. This procedure was repeated 5000 times to obtain a null distribution for each LC. We set the threshold for statistical significance at *P *<* *0.05 after false discovery rate correction.

To investigate the extent to which sensory symptom-related iFC and neurodevelopmental symptoms contributed to the character of each composite score, we computed Pearson correlation coefficients between ***X*** and ***L_X_***, as well as between ***Y*** and ***L_Y_***. A larger correlation coefficient for a sensory symptom-related iFC reflects a greater contribution to the sensory symptom-related iFC composite score. To estimate confidence intervals (CIs) for these correlation coefficients, we applied a bootstrapping method with 5000 iterations, generating 5000 samples with replacements from sensory symptom-related iFC and neurodevelopmental symptom data. *Z*-scores were computed by dividing the original correlation coefficients by the estimated standard deviation. These *Z*-scores were converted to *P*-values. We set the threshold for statistical significance at *P *<* *0.05 after false discovery rate correction.

To further examine whether the association between iFCs and neurodevelopmental symptoms (i.e. AQ and CAARS scores) was selective to sensory symptom-related iFCs, we conducted a control analysis based on a bootstrap framework. Briefly, we first randomly selected iFCs that were not included in the pool of sensory symptom-related iFCs. We then applied PLS-C with a permutation test to the bootstrapped dataset to assess the statistical associations between the resampled iFCs and neurodevelopmental symptoms. Repeating these procedures 5000 times yielded a *P*-value distribution for each LC. Actual *P*-values falling below the fifth percentile of the distributions were regarded as statistically significant (*P *<* *0.05).

### Data availability

The data analysed in the current study are available upon request.

## Results

### Intrinsic FC associated with sensory symptoms

**Figure 2:**
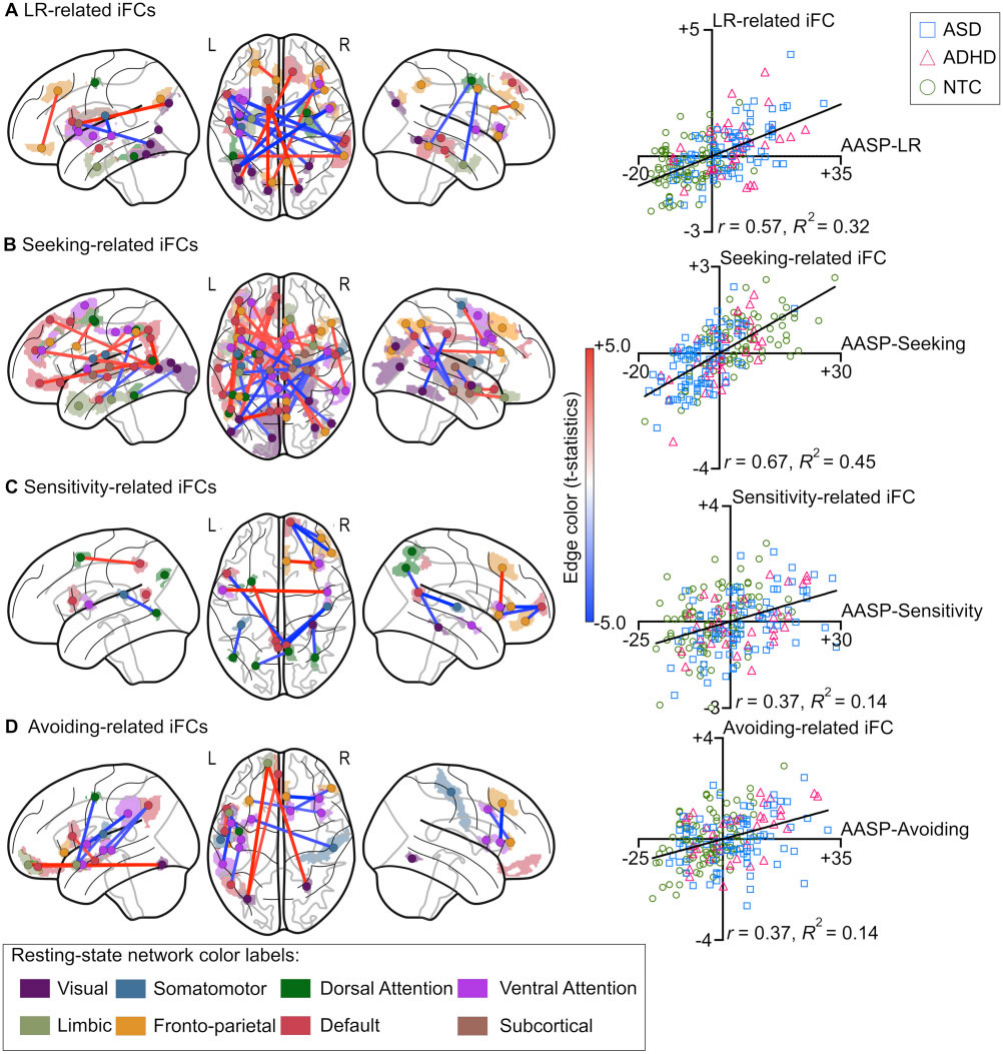
**Effects of sensory symptom severity on intrinsic brain functional connectivity (iFC).** The left column shows the iFC associated with each of four sensory symptom domains measured in the Adolescent/Adult Sensory Profile, such as low registration (A), sensation seeking (B), sensory sensitivity (C) and sensation avoiding (D). Age, handedness and head motion (median frame-wise displacement) were regressed out. The splotches and dots indicate the actual shapes and centre of coordinates of each region of interest. The colours of the splotches and dots represent the resting-state network to which the splotches and dots belong, while the line colour reflects the direction of the association between sensory symptoms and iFC. Red lines represent a positive association between sensory symptoms and iFC, while blue lines indicate a negative association. In the right column, scatter plots show the relationship between the summation of iFC strength for each sensory symptom score. For illustrative purposes, the correlation coefficients were converted to Z-scores using Fisher’s r-to-z transformation and integrated positive values and inverted negative values. Blue squares, red triangles and green circles represent individuals with autism spectrum disorder (ASD), those with attention-deficit/hyperactivity disorder (ADHD) and neurotypical controls (NTCs), respectively.

Our analyses revealed that each sensory symptom domain (i.e. low registration, sensation seeking, sensory sensitivity and sensation avoiding) was associated with a distinct iFC pattern. Low registration was associated with 23 instances of iFC. The network with the greatest number of instances of iFC related to low registration was the fronto-parietal network, followed by the visual network (Fig. 2A). Sensation seeking was associated with 39 instances of iFC (Fig. 2B). The default mode network was most frequently involved, followed by the ventral attention network (four instances of iFC on the right and four on the left). Sensory sensitivity was associated with 12 instances of iFC, and the default mode and dorsal attention networks were most frequently involved (Fig. 2C). Sensation avoidance was related to 13 instances of iFC (Fig. 2D). Only one instance of iFC was involved in two sensory symptom domains or more: the region between the right frontal supplementary medial and right insula was involved in both sensory sensitivity and sensation avoiding.

### Sensory symptom-related iFC associations between AQ and CAARS

**Figure 3:**
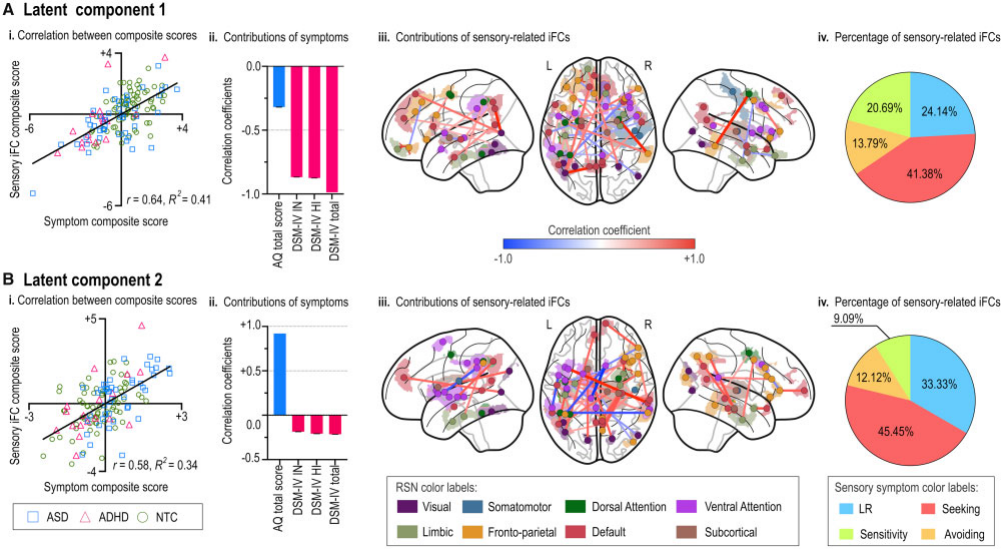
**Results of the partial least squares correlation (PLS-C) analysis.** We used a permutation test and the PLS-C analysis, controlling for the effects of sensory symptoms as well as other confounding factors (i.e. age, handedness and head motion), to identify two significant latent components (LC1: *q* < 0.001 and LC2: *q* < 0.001, false discovery rate-corrected). LC1 exhibited a significant association between sensory-related iFC and neurodevelopmental symptom-related composite scores (*r* = 0.64, *β* = 0.56, *R*^2^ = 0.41, *P* < 0.001) (**A**, i). Lower sensory symptom-related composite scores were negatively associated with both Autism Spectrum Quotient (AQ) total score and attention-deficit/hyperactivity disorder (ADHD) symptoms: AQ total score (*r* = −0.32, 95% CI = [−0.16, −0.44], *q* = 0.008), Conners’ Adult ADHD Rating Scale (CAARS) DSM-IV inattentive symptom severity (*r* = −0.87, 95% CI = [−0.82, −0.90], *q* < 0.001), CAARS DSM-IV hyperactive-impulsive symptom severity (*r* = −0.87, 95% CI = [−0.82, −0.92], *q* < 0.001) and DSM-IV ADHD total *T* score (*r* = −0.99, 95% CI = [−0.98, −0.99], *q* < 0.001) (**A**, ii). The edges represent the intrinsic brain functional connectivity (iFC) contributing to the sensory iFC composite score (**A**, iii). Low registration-, sensation seeking-, sensation avoiding- and sensory sensitivity-related iFC consisted of 24%, 41%, 14% and 21% of the sensory-related iFC composite score, respectively (**A**, iv). LC2 exhibited a statistically significant association between sensory-related iFC and neurodevelopmental symptom-related composite scores (*r* = 0.58, *β* = 0.86, *R*^2^ = 0.34, *P* < 0.001) (**B**, i). Greater symptom-related composite scores were positively correlated with AQ symptoms (*r* = 0.92, 95% CI = [0.88, 0.94], *q* < 0.001) and negatively correlated with CAARS scores, specifically DSM-IV hyperactive-impulsive symptom severity (*r* = −0.21, 95% CI = [−0.35, −0.05], *q* = 0.042), and ADHD total *T* score (*r* = −0.22, 95% CI = [−0.37, −0.06], *q* = 0.041), but not with DSM-IV inattentive symptom severity (*r* = −0.19, 95% CI = [−0.35, −0.02], *q* = 0.07 (**B**, ii)). (**B**, iii) The iFC that contributed to the sensory iFC composite score. Low registration-related and sensation seeking-related iFCs dominantly contributed to individual sensory-related iFC composite scores (**B**, iv).

PLS-C with a permutation test identified two significant LCs (LC1: *q *<* *0.001 and LC2: *q *<* *0.001, respectively). For LC1 [Fig. 3A(i)], the sensory symptom-related iFC composite score was positively associated with the neurodevelopmental symptom-related composite score (*r *=* *0.64, *β* = 0.56, *R*^2^ = 0.41, *P *<* *0.001). Pearson correlation analyses revealed that the neurodevelopmental symptom-related composite score was negatively associated with both total AQ score (*r* = −0.32, 95% CI = [−0.16, −0.44], *q *=* *0.008) and CAARS scores: DSM-IV inattentive symptom severity (*r* = −0.87, 95% CI = [−0.82, −0.90], *q *<* *0.001), DSM-IV hyperactive-impulsive symptom severity (*r* = −0.87, 95% CI = [−0.82, −0.92], *q *<* *0.001) and DSM-IV ADHD total *T* score (*r* = −0.99, 95% CI = [−0.98, −0.99], *q *<* *0.001) [Fig. 3A(ii)]. After thresholding bootstrapped *Z*-scores [Fig. 3A(iii)], a greater sensory symptom-related iFC composite score was positively associated with iFC from the default mode network to other networks, such as the visual network, while it was negatively associated with iFC between the ventral attention network and other networks, including the somatomotor and fronto-parietal networks. Sensation seeking-related iFC predominantly contributed to the sensory-related iFC composite score [Fig. 3A(iv)].

In terms of LC2 [Fig. 3B(i)], the sensory symptom-related iFC composite score was positively associated with neurodevelopmental symptom-related composite score (*r *=* *0.58, *β* = 0.86, *R*^2^ = 0.34, *P *<* *0.001). A greater neurodevelopmental symptom-related composite score was positively correlated with the AQ total score (*r *=* *0.92, 95% CI = [0.88, 0.94], *q *<* *0.001), but negatively correlated with the CAARS scores: DSM-IV hyperactive-impulsive symptom severity (*r* = −0.21, 95% CI = [−0.35, −0.05], *q *=* *0.042) and DSM-IV ADHD total *T* score (*r* = −0.22, 95% CI = [−0.37, −0.06], *q *=* *0.041). A greater sensory symptom-related iFC composite score was positively associated with iFC from the default mode network to other networks, such as the fronto-parietal, somatomotor and ventral attention networks, while it was negatively correlated with iFC between the fronto-parietal and dorsal/ventral attention networks [Fig. 3B(iii)]. Low registration-related and sensation seeking-related iFC dominantly contributed to individual sensory-related iFC composite scores [Fig. 3B(iv)].

Control analysis confirmed that, compared with the *P*-values of the resampled data, the *P*-values of the actual LC1 and LC2 fell below chance (LC1: *P *=* *0.003 and LC2: *P *<* *0.001). This suggests that the relationship between iFC and neurodevelopmental symptoms was selective for iFC associated with sensory symptoms.

## Discussion

We investigated the way in which sensory symptoms are associated with SCI and ADHD symptoms by analysing resting-state functional MRI data obtained from individuals with neurodevelopmental disorders and NTC. Dimensional analysis demonstrated that each of the four examined sensory symptom domains had a different iFC pattern. Focussing on the iFC patterns related to sensory symptoms, a multivariate data-driven analysis revealed that one set of iFC was predominantly associated with CAARS scores, while another set was mainly associated with AQ scores. When analysing the relationships between sensory symptom-related iFC and AQ and CAARS scores, we regressed out the effect of sensory symptoms to avoid a spurious correlation. Still, the relationship was selective to the neural bases of sensory symptoms. Both the relationship between sensory symptoms and iFC, as well as the iFC relationship between AQ and CAARS scores, were observed regardless of diagnosis. Further, we observed a substantial overlap across diagnostic groups. These findings suggest that continuity exists in sensory symptoms–neural circuits–ASD and ADHD symptom relationships across diagnoses, as well as highlighting the importance of sensory symptoms among symptoms of ASD and ADHD.

### Dimensional relationship between sensory symptoms and iFC

Consistent with the results of prior studies, the current study showed that individuals with ASD and those with ADHD obtained similar AASP scores in four domains (Clince *et al.*, 2016; Little *et al.*, 2018). By combining all of the individuals from the three diagnostic groups, the current study revealed that the neural correlates of each of the four sensory symptom domains were distinct. Notably, in all four domains, the scatter plots of sensory symptoms and iFC relationships showed substantial overlap of individuals across diagnostic groups (see Fig. 2), suggesting transdiagnostic homogeneity of neural correlates of sensory symptoms. The similarities in the slopes of sensory symptom-iFC relationships between people with ASD and those with ADHD support future examinations of the relationship between iFC and AQ and/or ADHD symptoms, regardless of diagnoses.

### Intrinsic brain functional connectivity with respect to ASD and ADHD symptoms

In line with prior studies (Tavassoli *et al.*, 2014; Bijlenga *et al.*, 2017), the AASP scores were correlated with both AQ and CAARS scores in the current participants, suggesting that iFC correlated with AASP scores can overlap with iFC associated with AQ or CAARS scores. Indeed, the neural correlates of sensory symptoms include not only the primary sensory areas but also the ventral attention and default mode networks, of which disturbances are often reported in the pathophysiology of ASD and ADHD (e.g. Sripada *et al.*, 2014; Padmanabhan *et al.*, 2017). As we aimed to examine the hierarchical relationships between the sensory symptoms-iFC-neurodevelopmental symptoms pathway, we regressed out sensory symptoms while examining the relationships between iFC and neurodevelopmental symptoms. Thus, the association between iFC and neurodevelopmental symptoms was not led by a correlation between sensory symptoms and neurodevelopmental symptoms.

Symptoms of ASD and ADHD have both shared and distinct neural mechanisms (Di Martino *et al.*, 2013; Kernbach *et al.*, 2018; Baribeau *et al.*, 2019). To focus on the neural mechanisms involved in both conditions, we adopted PLS-C. We found that the relationship between iFC and neurodevelopmental symptoms was selective to sensory symptom-related areas, reinforcing the important role of sensory symptoms in SCI and ADHD symptoms. Further, overlap in the relationship between iFC and neurodevelopmental symptoms across diagnostic groups implies the continuity of neurodevelopmental symptoms across individuals with ASD and those with ADHD from the perspective of the neural mechanisms of sensory symptoms.

## Limitations

The current results should be interpreted with caution. First, although the AASP is a frequently and internationally utilized questionnaire, we evaluated the sensory symptoms using self-report questionnaires. Future research should aim to objectively evaluate sensory symptoms. Examples of such objective measures could include behavioural tasks, conducted outside the scanner, that demand tactile, auditory or visual perception. Further, participants could complete a visual or auditory perception task during an fMRI session (Green *et al.*, 2019). Second, the three diagnostic groups were matched in terms of age, handedness and intelligence quotient. However, the number of individuals in each diagnostic group was not well balanced. As the current study was well powered and the contrast between diagnostic groups was not the main research question, we believe that our results were not influenced by the difference in the number of participants. Third, to increase biological homogeneity, we did not include female participants in the primary analysis. Indeed, several studies have emphasized sex-based iFC differences (Ypma *et al.*, 2016; Floris *et al.*, 2018). Although our supplementary analysis in which we included female participants produced similar results (Supplementary Figs 2 and 3), further studies are needed to generalize the current findings to women. In addition, as the primary aim of the current study was to examine the transdiagnostic features of the sensory–iFC–ASD and/or ADHD symptoms pathway, relationships across individuals with ASD and those with ADHD were examined. A future study with a larger sample size of NTCs would be helpful to examine the reported relationships in NTC. Fourth, we found that common symptoms were associated with common iFC, regardless of diagnosis. Although this could represent a step towards more precise medicine, for instance, treatments based on pathophysiology and not diagnosis, this was beyond the scope of the study. Fifth, we utilized the AQ score to represent SCI symptoms because of a high correlation between total and summed scores on the SCI subscales. However, AQ scores reflect both SCI and restricted interests and repetitive behaviours. Further, only one out of 50 questions in the AQ was about sensory over-reactivity. Thus, utilizing other psychological measures that purely focus on sensory perception, such as subdomains of ADOS, was ideal. However, this was hampered by several problems. First, as the primary aim of the current study was to investigate transdiagnostic features, the score needed to vary in non-ASD individuals. As the ADOS has a ‘floor effect’ in non-ASD individuals, it was not a practical choice. Further, even if the ADOS score varied in individuals with ADHD, only a small fraction of individuals with ADHD completed the ADOS evaluation because of financial restrictions and time constraints. As a practical approach, we examined the iFC-ASD symptoms association by replacing the AQ score with its SCI subscales, which showed substantially similar results. However, future studies that separate SCI from sensory symptoms in an objective and established manner are anticipated.

In conclusion, the current study demonstrates that each of the four sensory symptom domains (i.e. low registration, sensation seeking, sensory sensitivity and sensation avoidance) has different neural correlates, transdiagnostically observed in individuals with ASD, those with ADHD, and NTCs. Sensory symptom-related neural correlates were associated with both SCI and ADHD symptoms. Further, the association between iFC and SCI and ADHD symptoms was selective to the neural correlates of sensory symptoms. The current findings highlight the continuity of sensory–neural–ASD and ADHD symptoms relationships across the diagnostic boundary and demonstrate the importance of sensory symptoms in the pathophysiology of ASD and ADHD.

**Table 1:** Characteristics of adult male participants

	NTC (*n* = 96)	ASD (*n* = 78)	ADHD (*n* = 35)	**ANOVA or *t*-test** ^*^			
	Mean (SD)	Mean (SD)	Mean (SD)	*F* or *t*-statistics	df	*P*-Value	*Post hoc* test
Age	30.6 (6.7)	29.5 (6.7)	31.2 (9.4)	0.86	2, 206	0.42	
Handedness	89 (37)	86 (45)	74 (59)	1.64	2, 206	0.20	
FIQ	108 (7.6)	107 (14)	111 (13)	1.11	2, 168	0.33	
VIQ		111 (13)	112 (15)	−0.29	73	0.77	
PIQ		100 (16)	108 (15)	−1.67	73	0.10	
ADOS communication		4.0 (1.2)	0.6 (1.0)	8.15	65	<0.001	ADHD < ASD
ADOS reciprocal social interaction		7.1 (2.1)	1.5 (1.0)	8.25	65	<0.001	ADHD < ASD
ADOS-total		11 (3.0)	2.1 (1.0)	9.46	65	<0.001	ADHD < ASD
AQ							
SS	2.4 (2.2)	7.7 (2.1)	6.2 (2.9)	117.91	2, 191	<0.001	NTC < ADHD < ASD
AS	3.4 (1.4)	7.3 (1.8)	6.7 (2.2)	117.82	2, 191	<0.001	NTC < ADHD = ASD
ATD	4.3 (2.2)	5.7 (2.2)	4.9 (2.2)	8.59	2, 191	<0.001	NTC < ADHD = ASD
COM	1.8 (1.9)	7.2 (1.9)	6.3 (2.9)	150.94	2, 191	<0.001	NTC < ADHD = ASD
IMA	3.0 (1.6)	6.6 (2.0)	4.7 (2.3)	80.84	2, 191	<0.001	NTC < ADHD <ASD
Total score	14.9 (5.1)	34.4 (6.3)	28.7 (9.21)	214.54	2, 191	<0.001	NTC < ADHD <ASD
CAARS							
IM	46 (10)	64 (15)	73 (11)	53.75	2, 134	<0.001	NTC < ASD < ADHD
HR	50 (8.9)	54 (11)	64 (11)	15.16	2, 134	<0.001	NTC < ASD < ADHD
IE	42 (9.2)	58 (14)	64 (14)	39.81	2, 134	<0.001	NTC < ASD = ADHD
SC	46 (8.9)	61 (12)	61 (14)	33.53	2, 134	<0.001	NTC < ASD = ADHD
DSM-IV-IN	47 (7.8)	64 (14)	72 (14)	60.42	2, 134	<0.001	NTC < ASD < ADHD
DSM-IV-HI	47 (9.6)	57 (13)	65 (15)	23.05	2, 134	<0.001	NTC < ASD < ADHD
DSM-IV ADHD total	47 (8.9)	62 (13)	71 (13)	51.47	2, 134	<0.001	NTC < ASD < ADHD
ADHD index	45 (8.0)	65 (13)	69 (12)	67.45	2, 134	<0.001	NTC < ASD = ADHD
AASP							
Low registration	26 (6.8)	36 (9.0)	38 (8.3)	52.76	2, 206	<0.001	NTC < ASD = ADHD
Sensation seeking	40 (8.5)	32 (6.2)	37 (7.0)	25.19	2, 206	<0.001	ASD < ADHD = NTC
Sensory sensitivity	31 (8.1)	39 (11)	39 (10)	16.12	2, 206	<0.001	NTC < ASD = ADHD
Sensation avoiding	31 (7.5)	39 (11)	40 (11)	20.14	2, 206	<0.001	NTC < ASD = ADHD
Median FD (mm)	0.12 (0.04)	0.12 (0.06)	0.12 (0.04)	0.05	2, 206	0.95	
% of retained volumes	97.0 (5.0)	95.0 (7.2)	95.3 (7.8)	2.3	2, 206	0.1	

AASP = Adolescent/Adult Sensory Profile; ADHD = attention-deficit/hyperactivity disorder; ADOS = Autism Diagnostic Observation Schedule; AQ = Autism Spectrum Quotient; AS = attention switching/tolerance of change; ASD = autism spectrum disorder; ATD = attention to detail; CAARS = The Conners’ Adult ADHD Rating Scale; COM = communication skills; DSM = Diagnostic and Statistical Manual of Mental Disorders; DSM-IV-HI = DSM-IV Hyperactive-Impulsive symptoms; DSM-IV-IN = DSM-IV Inattentive symptoms; FD = frame-wise displacement; FIQ = full-scale intelligence quotient; HR = hyperactivity/restlessness; IE = impulsivity/emotional lability; IM = inattention/memory problems; IMA = imagination; NTC = neurotypical control; PIQ = performance intelligence quotient; SC = problems of self-concept; SD = standard deviation; SS = social skills; VIQ = verbal intelligence quotient.

* We conducted *t*-test only when scores for two groups were available.

## Supplementary material

Supplementary material is available at *Brain Communications* online.

## Funding

This work was supported by The Japan Society for the Promotion of Science KAKENHI (19K03370 and 19H04883 to T.I. and 18K15493 to Y.Y.A.); Takeda Science Foundation; the SENSHIN Medical Research Foundation (to Y.Y.A.). This work was also supported by the Japan Agency for Medical Research and Development (grant numbers JP19dm0307008 to R.-I.H., JP19dm0307026 to T.I., and JP19dm0307001 and 20dm0307105 to M.N.).

## Competing interests

The authors declare no conflict of interest.

## Supplementary Material

fcaa186_Supplementary_Data

## Abbreviations

AASP =: Adolescent/Adult Sensory Profile
ADHD =: attention-deficit/hyperactivity disorder
ADOS =: Autism Diagnostic Observation Schedule
AQ =: Autism Spectrum Quotient
ASD =: autism spectrum disorder
CAARS =: Conners’ Adult ADHD Rating Scale
CI =: confidence interval
DSM =: Diagnostic and Statistical Manual of Mental Disorders
FD =: frame-wise displacement
iFC =: intrinsic brain functional connectivity
LC =: latent component
NTC =: neurotypical control
PLS-C =: partial least squares correlation
SCI =: social communication impairment
