# Supplementary material for: Neural correlates of shared sensory symptoms in autism and attention-deficit/hyperactivity disorder
Source: Brain Commun. 2020 Nov 2;2(2):fcaa186. doi: 10.1093/braincomms/fcaa186 (PMC7753051; doi:10.1093/braincomms/fcaa186)
Supplement: fcaa186_Supplementary_Data [file fcaa186_supplementary_data.docx]

**Supplementary information for “Neural correlates of shared sensory symptoms in autism and attention-deficit/hyperactivity disorder”**

By Itahashi et al.

**Supplementary Methods**

**Supplementary analyses**

**Comparison of interaction between sensory symptom-related iFC and neurodevelopmental symptoms between diagnostic groups**

We conducted supplementary analyses to examine the interaction effects between sensory symptom-related iFC and neurodevelopmental symptoms across diagnostic groups (i.e., ASD and ADHD groups). Here, we used the AQ total score and the CAARS DSM-IV total score as representative measures for ASD and ADHD symptoms, respectively. We included the four sensory symptoms as nuisance covariates in a general linear model. Permutation tests with 5,000 iterations were performed to identify the statistically significant interaction effects between the diagnostic groups and neurodevelopmental symptoms. We set the threshold for statistical significance at *P* < 0.05 after FDR correction.

**Replication of associations by replacing the AQ total score with the AQ subscales that measure SCI**

As the AQ total scores were well correlated with the scores for subscales measuring SCI (i.e, social skills and communication skills, see Methods section), we regarded the AQ score to represent SCI symptom severity in the current study. However, the AQ total score included a number of questions regarding sensory symptoms. Thus, to avoid circular theory, we repeated the PLS-C analysis by replacing the AQ total score with the sum of the two AQ subscales regarding SCI. The PLS-C analysis procedure was the same as described above.

**Associations between sensory-related intrinsic brain functional connectivity (iFC) and neurodevelopmental symptoms while including female participants**

In the main analyses, we excluded 42 female participants to increase the biological homogeneity of the dataset. To test the generalizability of our findings, we repeated a series of analyses while including the female participants. The overall procedures were the same as described in the main manuscript. We included biological sex as an additional nuisance covariate in the general linear model, as well as in the partial least squares correlation (PLS-C) analysis.

**Supplementary Results**

**Supplementary analyses**

**Interaction between sensory symptom-related iFC and neurodevelopmental symptoms among diagnostic groups**

As shown in Fig. S2, permutation tests identified iFC between the right superior temporal gyrus and the triangular part of right inferior frontal gyrus that exhibited interaction effects between clinical diagnosis and the AQ total score (*q* = 0.008).

**Replication of associations by replacing the AQ total score with the AQ subscales that measure SCI**

Consistent with the main analysis, PLS-C analysis identified two significant latent components (LC1: *q* < 0.001 and LC2: *q* < 0.001). As shown in Fig. S3A (i), LC1 showed a significant association between sensory-related iFC and neurodevelopmental symptom-related composite scores (*r* = 0.64, *β* = 0.55, *R*^2^ = 0.41, *p* < 0.001). Pearson correlation analyses revealed that neurodevelopmental symptom-related composite scores were negatively correlated with AQ SCI score (*r* = -0.30, 95%CI = [-0.16, -0.43], *q* < 0.001) and CAARS:DSM-IV inattentive symptom severity (*r* = -0.88, 95%CI = [-0.83, -0.91], *q* < 0.001), DSM-IV hyperactive-impulsive symptom severity (*r* = -0.87, 95%CI = [-0.81, -0.91], *q* < 0.001), and DSM-IV ADHD total *T* score (*r* = -0.99, 95%CI = [-0.98, -0.99], *q* < 0.001) (Fig. S3A (ii)). The pattern of contribution of sensory-related iFC to the corresponding composite score was similar (Fig. S3A (iii)). Seeking-related iFC dominantly contributed to sensory-related iFC composite scores (Fig. S3A (iv)).

For LC2 (Fig. S3B (i)), sensory-related iFC composite scores were significantly correlated with neurodevelopmental symptom-related composite scores (*r* = 0.58, *β* = 0.91, *R*^2^ = 0.34, *p* < 0.001). Neurodevelopmental symptom-related composite scores were positively correlated with AQ SCI score (*r* = 0.94, 95%CI = [0.90, 0.96], *q* < 0.001) and negatively correlated with CAARS:DSM-IV hyperactive-impulsive symptom severity (*r* = -0.23, 95%CI = [-0.38, -0.07], *q* = 0.008), except for the following scales: CAARS:DSM-IV inattentive symptom severity (*r* = -0.01, 95%CI = [-0.17, 0.15], *q* = 0.91) and DSM-IV ADHD total *T* score (*r* = -0.12, 95%CI = [-0.27, 0.03], *q* = 0.16) (Fig. S3B (ii)). Low-registration and seeking-related iFC predominantly contributed to the sensory-related iFC composite score (Fig. S3B (iv)). These data indicate that the main findings were not derived from scores associated with sensory symptoms in the AQ total score.

**Association between sensory-related iFC and neurodevelopmental symptoms while including female participants**

As shown in Fig. S4, statistical analyses identified a distinct pattern of iFC associated with each of the four sensory symptoms (low registration: 18 instances of iFC, sensation seeking: 36 instances of iFC, sensory sensitivity: 10 instances of iFCs, and sensation avoidance: 21 instances of iFCs).

Consistent with the main analysis, PLS-C analysis identified two significant latent components (LC1: *q* < 0.001 and LC2: *q* = 0.023, FDR-corrected). As shown in Fig. S5A (i), LC1 showed a significant association between sensory-related iFC and neurodevelopmental symptom-related composite scores (*r* = 0.59, *β* = 0.48, *R*^2^ = 0.35, *p* < 0.001). Pearson correlation analyses revealed that neurodevelopmental symptom-related composite scores were negatively correlated with both AQ total score (*r* = -0.42, 95%CI = [-0.29, -0.53], *q* < 0.001) and CAARS scores, including DSM-IV inattentive symptom severity (*r* = -0.86, 95%CI = [-0.81, -0.89], *q* < 0.001), DSM-IV hyperactive-impulsive symptom severity (*r* = -0.85, 95%CI = [-0.80, -0.90], *q* < 0.001), and DSM-IV ADHD total *T* score (*r* = -0.97, 95%CI = [-0.95, -0.98], *q* < 0.001) (Fig. S5A (ii)). Seeking-related and avoiding-related iFC dominantly contributed to sensory-related iFC composite scores (Fig. S5A (iv)).

For LC2 (Fig. S5B (i)), sensory-related iFC composite scores were significantly correlated with neurodevelopmental symptom-related composite scores (*r* = 0.44, *β* = 0.60, *R*^2^ = 0.19, *p* < 0.001). Neurodevelopmental symptom-related composite scores were positively correlated with the AQ total score (*r* = 0.77, 95%CI = [0.68, 0.82], *q* < 0.001), and negatively associated with CAARS scores, including DSM-IV inattentive symptom severity (*r* = -0.40, 95%CI = [-0.24, -0.53], *q* < 0.001), DSM-IV hyperactive-impulsive symptom severity (*r* = -0.46, 95%CI = [-0.32, -0.58], *q* < 0.001), and DSM-IV ADHD total *T* score (*r* = -0.48, 95%CI = [-0.34, 0.59], *q* < 0.001) (Fig. S5B (ii)). Low-registration and seeking-related iFC predominantly contributed to sensory-related iFC composite scores (Fig. S5B (iv)).

**Supplementary Figures:**


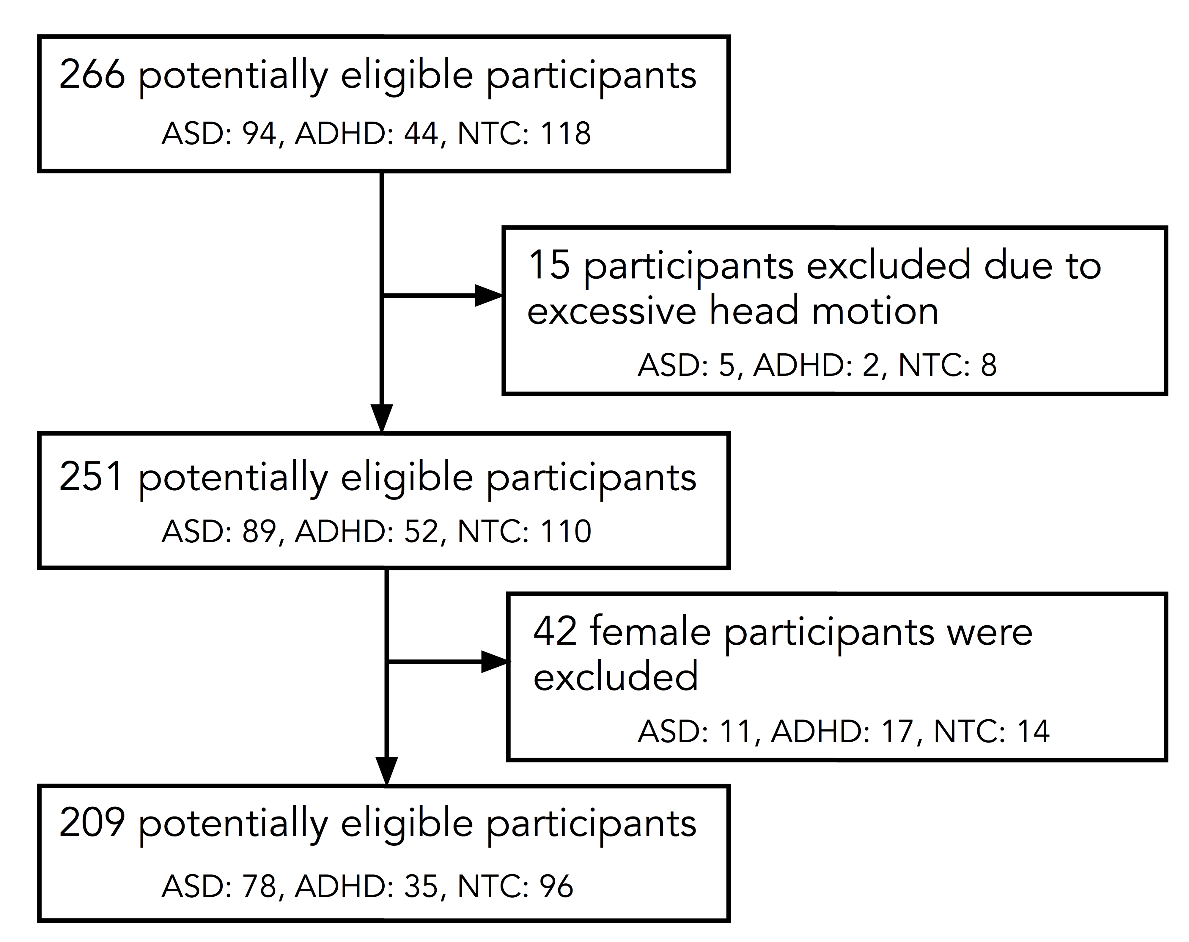


**Figure S1: Flow chart of participant inclusion/exclusion.**

From 266 potentially eligible participants, we first excluded 15 participants because of excessive head motion during the scans. Then, to improve the biological homogeneity of the sample, we further excluded 42 female participants. Finally, data from 209 participants were analyzed in this study.


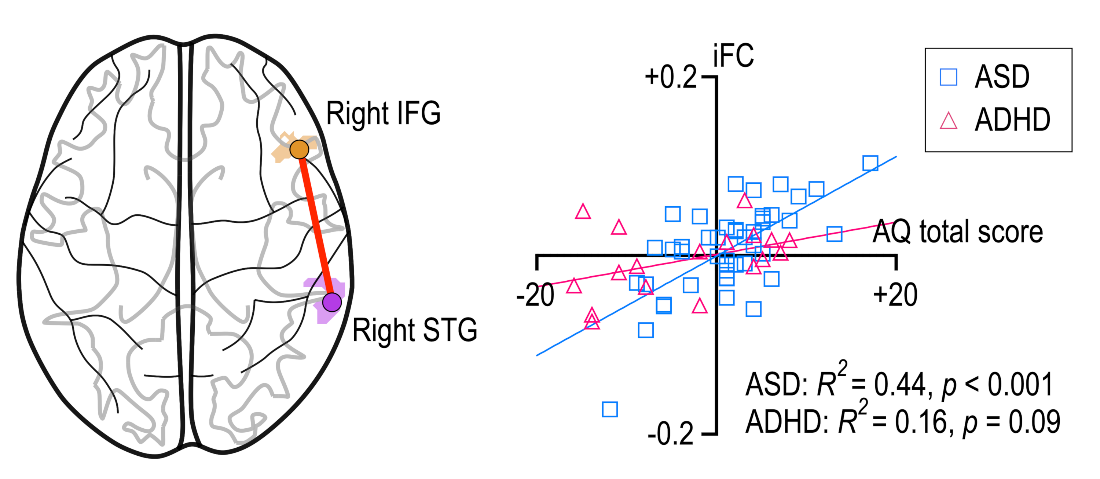


**Figure S2:** **Interaction effects between diagnostic groups and Autism Spectrum Quotient scores with respect to sensory-related iFC.**

Statistical analyses controlling for age, handedness, head motion, and sensory symptoms identified that one instance of iFC between the right superior temporal gyrus (STG) and the triangular part of the right inferior frontal gyrus (IFG) exhibited an interaction effect between diagnostic groups and the Autism Spectrum Quotient (AQ) total score (*q* = 0.008). Scatter plot shows associations between the strength of iFC and AQ total score in the ASD group (blue) and the ADHD group (red). In the scatter plot, nuisance covariates were regressed out from the iFC and the AQ total score.


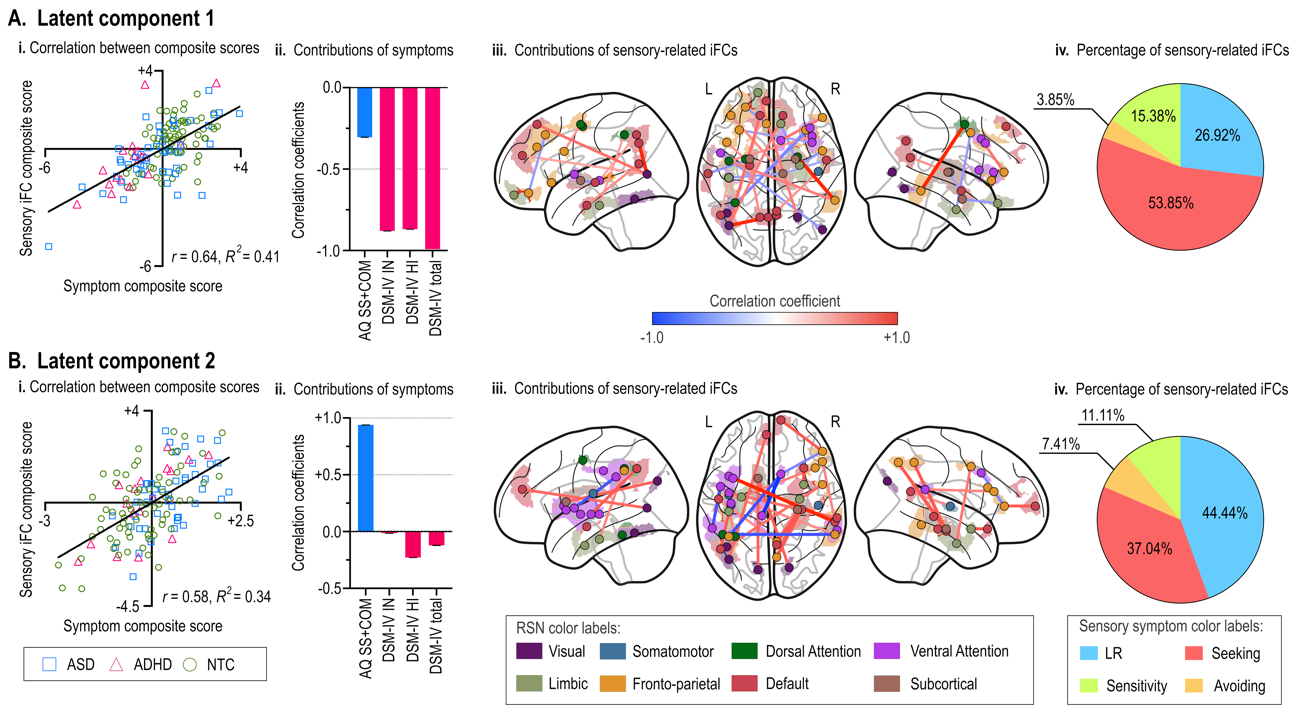


**Figure S3: Results of partial least squares correlation (PLS-C) analysis with the summation of social skills and communication as the index of social communication impairment (SCI).**

PLS-C controlling for the effects of sensory symptoms as well as other confounding factors (i.e., age, handedness, and head motion) identified two significant latent components (LC1: *q* < 0.001 and LC2: *q* < 0.001, FDR-corrected). LC1 exhibited a significant association between sensory-related iFC and neurodevelopmental symptom-related composite scores (*r* = 0.64, *β* = 0.55, *R*^2^ = 0.41, *p* < 0.001) (A-i). Neurodevelopmental symptom-related composite scores were negatively associated with both AQ SCI scores (*r* = -0.30, 95%CI = [-0.16, -0.43], *q* < 0.001) and Conners’ Adult ADHD Rating Scale (CAARS) scores, including DSM-IV inattentive symptom severity (*r* = -0.88, 95%CI = [-0.83, -0.91], *q* < 0.001), DSM-IV hyperactive-impulsive symptom severity (*r* = -0.87, 95%CI = [-0.81, -0.91], *q* < 0.001), and DSM-IV ADHD total *T* score (*r* = -0.99, 95%CI = [-0.98, -0.99], *q* < 0.001) (A-ii). The edges show the intrinsic brain functional connectivity (iFC) contributing to the sensory iFC composite score (A-iii). Low registration-, sensation seeking-, sensation avoiding-, and sensory sensitivity-related iFC comprised around 27%, 54%, 4%, and 15% of the sensory-related iFC composite score, respectively (A-iv). LC2 exhibited a statistically significant association between sensory-related iFC and neurodevelopmental symptom-related composite scores (*r* = 0.58, *β* = 0.91, *R*^2^ = 0.34, *p* < 0.001) (B-i). Greater symptom-related composite scores were positively correlated with AQ SCI scores (*r* = 0.94, 95%CI = [0.90, 0.96], *q* < 0.001) and negatively correlated with CAARS scores, including DSM-IV hyperactive-impulsive symptom severity (*r* = -0.23, 95%CI = [-0.38, -0.07], *q* = 0.008) but not CAARS DSM-IV inattentive symptom severity (*r* = -0.01, 95%CI = [-0.17, 0.15], *q* = 0.91) or DSM-IV ADHD total *T* score (*r* = -0.12, 95%CI = [-0.27, 0.03], *q* = 0.16) (B-ii). (B-iii) shows the contribution of iFC to the sensory iFC composite scores. Low registration-related and sensation seeking-related iFC dominantly contributed to individual sensory-related iFC composite scores (B-iv).


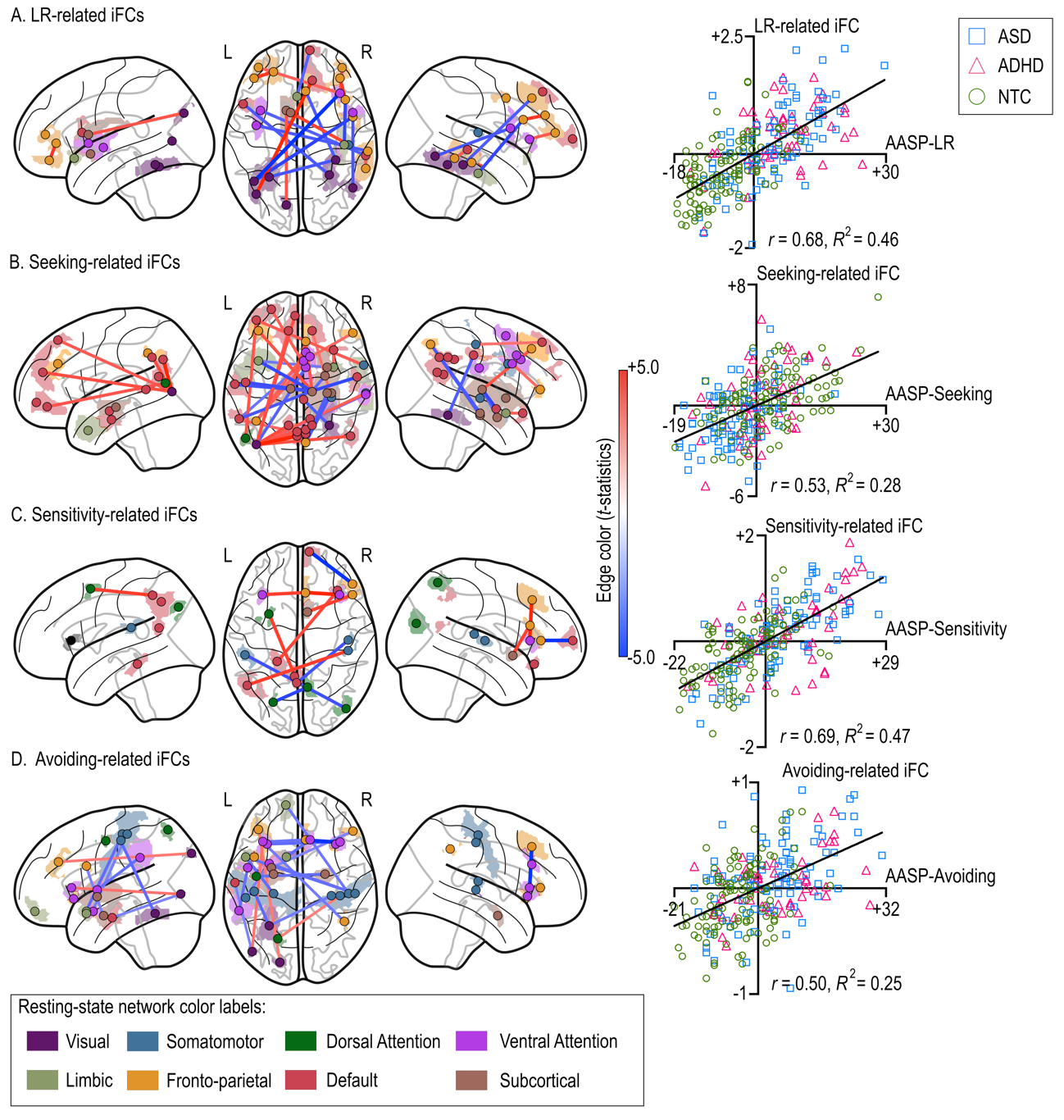


**Figure S4:** **Effects of sensory symptom severity on intrinsic brain functional connectivity while including female participants.**

The left column shows the intrinsic brain functional connectivity (iFC) associated with each of the four domains of sensory symptoms measured in the Adolescent/Adult Sensory Profile. Age, handedness, head motion (median frame-wise displacement), and biological sex were regressed out. Four sensory symptoms were associated with distinct sets of iFC (low registration: 18 sets of iFC, sensation seeking: 36 sets of iFC, sensory sensitivity: 10 sets of iFCs, and sensation avoidance: 21 sets of iFCs) (A-D in the left column). In the right column, scatter plots show the relationship between the summation of iFC strength for each result and sensory symptom score. For illustrative purposes, correlation coefficients were converted to *z*-scores using Fisher’s *r*-to-*z* transformation and then integrate positive values and inverted negative values. The splotches and dots in the left column indicate the actual shapes and center of coordinates for each region of interest (ROI). The colors of the splotches and dots represent the resting-state network to which the splotches and dots belong, while the line colors reflect the direction of the association between sensory symptoms and iFC. The red line represents a positive correlation between sensory symptoms and iFC, while the blue line indicates a negative correlation. Blue squares, red triangles, and green circles represent individuals with autism spectrum disorder (ASD), those with attention-deficit/hyperactivity disorder (ADHD), and neurotypical controls (NTC), respectively.

**
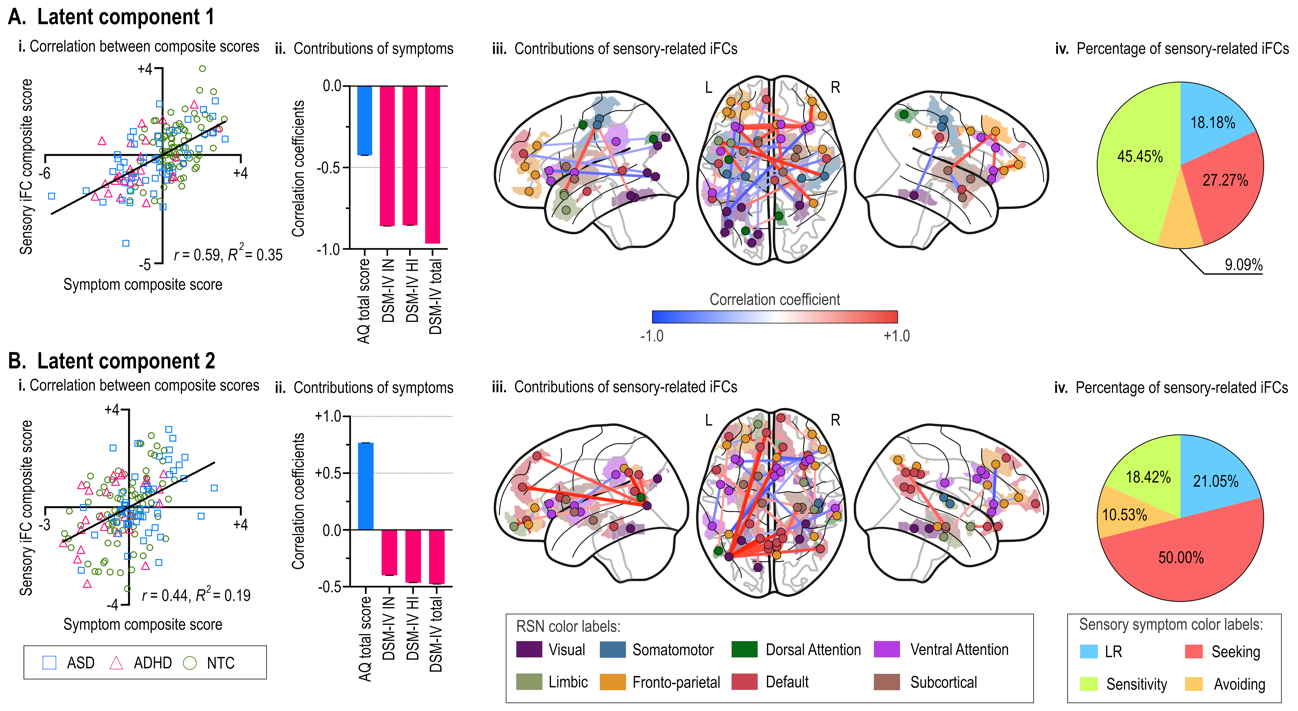
**

**Figure S5: Results of partial least squares correlation (PLS-C) analysis while including female participants.**

PLS-C controlling for the effects of sensory symptoms as well as other confounding factors (i.e., age, handedness, head motion, and biological sex) identified two significant latent components (LC1: *q* < 0.001 and LC2: *q* = 0.023, FDR-corrected). LC1 exhibited a significant association between sensory-related iFC and neurodevelopmental symptom-related composite scores (*r* = 0.59, *β* = 0.48, *R*^2^ = 0.35, *p* < 0.001) (A-i). Neurodevelopmental symptom-related composite scores were negatively associated with both AQ total scores (*r* = -0.42, 95%CI = [-0.29, -0.53], *q* < 0.001) and Conners’ Adult ADHD Rating Scale (CAARS), including DSM-IV inattentive symptom severity (*r* = -0.86, 95%CI = [-0.81, -0.89], *q* < 0.001), DSM-IV hyperactive-impulsive symptom severity (*r* = -0.85, 95%CI = [-0.80, -0.90], *q* < 0.001), and DSM-IV ADHD total *T* score (*r* = -0.97, 95%CI = [-0.95, -0.98], *q* < 0.001) (A-ii). The edges show the intrinsic brain functional connectivity (iFC) contributing to the sensory iFC composite score (A-iii). Avoiding-related and seeking-related iFC contributed to the sensory-related iFC composite score (A-iv). LC2 exhibited a statistically significant association between sensory-related iFC and neurodevelopmental symptom-related composite scores (*r* = 0.44, *β* = 0.60, *R*^2^ = 0.19, *p* < 0.001) (B-i). Greater symptom-related composite scores were positively correlated with AQ total scores (*r* = 0.77, 95%CI = [0.68, 0.82], *q* < 0.001), and negatively associated with CAARS scores, including DSM-IV inattentive symptom severity (*r* = -0.40, 95%CI = [-0.24, -0.53], *q* < 0.001), DSM-IV hyperactive-impulsive symptom severity (*r* = -0.46, 95%CI = [-0.32, -0.58], *q* < 0.001), and DSM-IV ADHD total *T* score (*r* = -0.48, 95%CI = [-0.34, 0.59], *q* < 0.001) (B-ii). (B-iii) shows the iFC contributing to the sensory iFC composite score. Low registration-related, sensation seeking-related, and sensitivity-related iFC dominantly contributed to individual sensory-related iFC composite scores (B-iv).
